# Effects of Household Cooking Methods on the Nutritional, Phytochemical and Functional Properties of *Cucurbita maxima* Leaves

**DOI:** 10.3390/molecules31162740

**Published:** 2026-08-07

**Authors:** Małgorzata Stryjecka, Tomasz Cebulak, Anna Kiełtyka-Dadasiewicz, Ewa Stamirowska-Krzaczek, Marzena Tomaszewska, Barbara Krochmal-Marczak, Izabela Betlej

**Affiliations:** 1 Institute of Human Nutrition and Agriculture, The University College of Applied Sciences in Chełm, 22-100 Chelm, Poland; ekrzaczek@panschelm.edu.pl (E.S.-K.); mtomaszewska@panschelm.edu.pl (M.T.); 2 Department of Food Technology and Human Nutrition, Institute of Food Technology and Nutrition, College of Natural Sciences, University of Rzeszów, 35-601 Rzeszów, Poland; 3 Department of Plant Production Technology and Commodity Sciences, University of Life Sciences, 20-950 Lublin, Poland; anna.kieltyka-dadasiewicz@up.edu.pl; 4 Department of Plant Production and Food Safety, The University College of Applied Sciences in Krosno, 38-400 Krosno, Poland; barbara.marczak@pans.krosno.pl; 5 Institute of Wood Sciences and Furniture, Warsaw University of Life Sciences—SGGW, 159 Nowoursynowska St., 02-776 Warsaw, Poland; izabela_betlej@sggw.edu.pl

**Keywords:** *Cucurbita maxima* leaves, cooking methods, phenolic compounds, antioxidant activity, antinutritional factors, α-amylase inhibition, functional foods

## Abstract

Pumpkin leaves (*Cucurbita maxima* L.) are an underutilized leafy vegetable that represents a rich source of nutrients and bioactive compounds. This study assessed the effects of four common household cooking methods—boiling, steaming, microwaving, and stir-frying—on the nutrient composition, amino acid profile, phenolic compounds, carotenoids, antioxidant potential, antinutritional factors, and α-amylase inhibitory activity of young pumpkin leaves. Steaming resulted in the highest apparent retention of phenolics, flavonoids, and carotenoids. Microwave treatment also maintained relatively high levels of phytochemicals, whereas boiling caused the greatest losses of bioactive compounds. Steaming also showed the strongest α-amylase inhibitory activity. Boiling, on the other hand, was the most effective method for reducing antinutritional compounds. The results demonstrate that culinary processing significantly affects the nutritional and functional quality of pumpkin leaves. Collectively, the results suggest that steaming was the most effective method for preserving nutrients and bioactive compounds, whereas boiling was more effective in reducing antinutritional compounds. These findings provide practical evidence-based recommendations for optimizing household cooking methods according to specific nutritional objectives.

## 1. Introduction

Leafy vegetables are widely recognized as essential components of healthy diets due to their high content of vitamins, minerals, dietary fiber, and diverse phytochemicals associated with the prevention of chronic non-communicable diseases. Epidemiological evidence indicates that regular consumption of green leafy vegetables is associated with a reduced risk of cardiovascular diseases, type 2 diabetes, obesity, and several forms of cancer, largely owing to their antioxidant, anti-inflammatory, and metabolic regulatory properties [1,2,3]. Consequently, increasing attention has been directed toward identifying underutilized plant resources that may contribute to nutritional security, dietary diversification, and the development of sustainable food systems.

Among such resources, pumpkin (*Cucurbita* spp.) has attracted considerable scientific interest because of its nutritional value and abundance of bioactive compounds distributed throughout different plant organs, including fruits, seeds, peels, flowers, and leaves [4,5]. Although pumpkin fruits are widely consumed worldwide, the leaves remain largely underutilized despite their long history of use as traditional vegetables in many African, Asian, and Latin American regions [6,7,8]. In recent years, pumpkin leaves have gained increasing attention as a potentially valuable source of nutrients and health-promoting phytochemicals capable of supporting both food security and functional food development [9,10].

Pumpkin leaves are characterized by a favorable nutritional composition, containing substantial amounts of protein, dietary fiber, minerals, carotenoids, and other bioactive constituents [10,11]. In addition to their nutritional value, these leaves contain a diverse array of phenolic compounds, particularly phenolic acids and flavonoids, which have been identified as important contributors to their biological activity [10,11]. Such compounds have attracted significant interest because of their antioxidant properties and their potential involvement in physiological processes associated with inflammation, oxidative stress, and glucose metabolism [12,13].

Several phenolic compounds commonly identified in pumpkin leaves, including caffeic acid, chlorogenic acid, rutin, quercetin derivatives, and kaempferol glycosides, have been reported to exhibit antioxidant and enzyme-modulating activities [10,13]. Experimental studies on plant-derived polyphenols suggest that these compounds may interact with carbohydrate-hydrolyzing enzymes, including α-amylase, thereby contributing to the regulation of postprandial glucose responses [13,14]. Dietary inhibition of α-amylase has attracted considerable interest as a nutritional strategy for reducing postprandial hyperglycemia. Despite the growing body of evidence concerning the composition and biological properties of pumpkin leaves, important knowledge gaps remain regarding the effects of culinary processing on their nutritional and functional quality. Thermal processing is an indispensable step in the preparation of leafy vegetables because it improves palatability, digestibility, and microbiological safety. At the same time, cooking may profoundly influence nutrient retention, phytochemical composition, antioxidant potential, and biological activity through mechanisms including thermal degradation, oxidation, leaching, and structural modification of plant tissues [15,16,17]. The magnitude and direction of these changes are strongly dependent on the cooking method employed.

Previous investigations on pumpkin and other leafy vegetables have demonstrated that boiling, steaming, microwaving, and frying can differentially affect phenolic compounds, carotenoids, antioxidant activity, and antinutritional factors [10,15,16,17]. However, most available studies have focused on selected compositional attributes, individual classes of phytochemicals, or isolated biological activities. Existing studies have largely focused on isolated quality attributes or individual cooking methods, making it difficult to evaluate the overall consequences of culinary processing for the health-promoting potential of this underutilized vegetable.

To the best of our knowledge, no previous study has comprehensively integrated proximate composition, amino acid profiling, characterization of individual phenolic acids and flavonoids, antinutritional factors, antioxidant activity, and inhibition of α-amylase within a single experimental framework to evaluate the effects of boiling, steaming, microwaving, and stir-frying on *C*. *maxima* leaves. By simultaneously examining nutrient retention, phytochemical stability, reduction in antinutritional compounds, antioxidant capacity, and α-amylase inhibitory activity, the present study provides a multidimensional assessment of cooking-induced quality changes that extends beyond conventional compositional analyses.

Therefore, the aim of this study was to evaluate the effects of four commonly used household cooking methods—boiling, steaming, microwaving, and stir-frying—on the proximate composition, amino acid profile, phytochemical composition, antioxidant activity, antinutritional factors, and inhibitory activity against α-amylase in pumpkin (*C. maxima* L.) leaves. We hypothesized that cooking methods involving limited contact with water, particularly steaming and microwaving, would better preserve nutrients and bioactive compounds than conventional boiling, resulting in superior apparent retention of antioxidant and enzyme inhibitory properties. The findings are expected to provide evidence-based recommendations for optimizing household preparation practices while maximizing the nutritional and functional value of pumpkin leaves as an underutilized leafy vegetable.

## 2. Results and Discussion

### 2.1. Proximate Composition of Raw and Processed Pumpkin Leaves

Because changes in moisture substantially influence nutrient concentrations after thermal processing, subsequent comparisons are based primarily on dry weight values and represent apparent retention rather than true retention.

The proximate composition of raw and processed *C. maxima* leaves expressed on both a fresh-weight (FW) and dry-weight (DW) basis is presented in Table 1. Moisture content ranged from 80.25% in stir-fried leaves to 88.10% in boiled samples. The higher moisture content observed after boiling can be attributed to water uptake and tissue hydration during cooking, whereas the lower moisture level in stir-fried leaves is consistent with intensive moisture evaporation caused by high-temperature dry-heat processing. Similar trends have been reported in vegetables subjected to different cooking methods, where boiling generally increases tissue water content, while frying and stir-frying promote water loss through evaporation [18,19]. The observed changes may be explained by heat-induced disruption of parenchyma cell walls and partial solubilization of middle lamella pectins, which increase membrane permeability. Consequently, water-soluble minerals, free amino acids and other low-molecular-weight compounds may diffuse into the cooking medium. In contrast, steaming minimizes direct contact between plant tissues and water, thereby reducing diffusional losses while preserving cellular integrity to a greater extent.

Therefore, further discussion is based primarily on dry weight values. The ash content on a fresh-weight basis ranged from 1.76 g/100 g in boiled leaves to 2.42 g/100 g in stir-fried samples. Nevertheless, after normalization to dry matter, ash concentrations were relatively similar among raw, microwaved, steamed, and boiled leaves (14.79–15.49 g/100 g DW). The lower ash value observed in stir-fried leaves (12.25 g/100 g DW) suggests that the apparent increase recorded on a fresh-weight basis was largely attributable to moisture loss rather than enhanced mineral retention. The lower ash content observed after boiling is consistent with the leaching of water-soluble minerals into the cooking medium, a phenomenon widely reported in cooked vegetables and leafy greens. Mineral losses during boiling are primarily attributed to diffusion into the surrounding water and are influenced by cooking time, temperature, and the water-to-plant ratio [16,20].

Protein content exhibited a similar pattern. On a fresh-weight basis, stir-fried leaves contained the highest protein concentration (4.92 g/100 g), whereas boiled samples showed the lowest value (3.78 g/100 g). However, dry weight calculations revealed remarkably similar protein concentrations in raw, boiled, microwaved, and steamed leaves (31.16–31.76 g/100 g DW). Taken together, these findings indicate that thermal processing affects protein concentration. The lower protein concentration observed in stir-fried leaves (24.91 g/100 g DW) is more likely attributable to the increased lipid content resulting from oil absorption during frying, which altered the relative contribution of other nutrients to the dry matter composition rather than reflecting substantial protein losses [21,22,23].

The most pronounced effect of stir-frying was observed for fat content. Whereas raw leaves contained only 0.80 g/100 g FW (5.80 g/100 g DW), stir-fried samples reached 2.85 g/100 g FW and 14.43 g/100 g DW. This increase is attributable to oil uptake during frying, a phenomenon widely documented in fried foods and associated with moisture loss and subsequent oil absorption into the plant matrix [24,25]. In contrast, boiling, steaming, and microwaving caused only minor changes in lipid concentration, indicating limited loss or degradation of endogenous lipids under these processing conditions [15,16]. It should be noted that the use of extra virgin olive oil during stir-frying may have contributed to the measured lipid content and may have influenced the phenolic composition and antioxidant activity of the samples. Therefore, the results obtained for stir-fried leaves should be interpreted with this potential contribution of the cooking medium in mind.

Dietary fiber content ranged from 2.58 to 3.48 g/100 g FW and from 17.62 to 22.63 g/100 g DW. On a dry matter basis, steamed and microwaved leaves retained fiber levels comparable to those of raw leaves, whereas boiling and stir-frying resulted in lower values. The reduction observed after boiling may be attributed to the partial solubilization of fiber components, particularly pectic substances and hemicelluloses, followed by leaching into the cooking water. Similar effects have been reported for spinach, amaranth, and other leafy vegetables subjected to thermal processing [15,20].

Carbohydrate content demonstrated the greatest variation among treatments. On a dry matter basis, boiled leaves contained 26.64 g/100 g DW, while stir-fried samples reached 30.78 g/100 g DW. The elevated carbohydrate proportion observed after stir-frying should not be interpreted as carbohydrate enrichment. Rather, it reflects changes in the relative composition of dry matter resulting from moisture loss and increased fat incorporation. Such proportional shifts are commonly observed when vegetables are subjected to high-temperature cooking methods [15].

To better distinguish between concentration effects caused by moisture loss and actual compositional changes, results were additionally expressed on a dry-weight basis. The relatively small differences observed among boiling, microwaving and steaming treatments after moisture correction indicate that many apparent changes on a fresh-weight basis were primarily associated with variations in water content rather than substantial alterations in nutrient concentration.

### 2.2. Effect of Culinary Processing on the Amino Acids Composition of Cucurbita maxima Leaves Expressed on a Dry-Weight (DW) Basis (mg/g DW)

The amino acid composition of *C. maxima* leaves was significantly influenced by the applied culinary treatments (Table 2). Glutamic acid, aspartic acid, leucine, lysine, and arginine were the predominant amino acids in both raw and processed samples, suggesting that pumpkin leaves constitute a valuable source of dietary protein. Thermal processing generally reduced amino acid concentrations, although the magnitude of losses depended on the cooking method.

Thermal treatment may also induce protein unfolding and partial denaturation, exposing amino acid residues to oxidation and Maillard-type reactions. Sulfur-containing amino acids, particularly cysteine and methionine, are especially susceptible because sulfhydryl groups are readily oxidized during heating. Furthermore, free amino acids may diffuse into the cooking medium during boiling owing to their high water solubility. The combined effects of thermal degradation, oxidation, and leaching provide a plausible explanation for the greater amino acid losses recorded after boiling compared with steaming.

Steaming resulted in the highest apparent retention of both essential and non-essential amino acids on a dry-weight basis, with most compounds remaining above 90% of their original concentrations. Microwave treatment also preserved amino acids effectively, whereas boiling caused the greatest reductions. The substantial losses observed after boiling are likely attributable to the leaching of soluble amino acids into the cooking medium and to thermal degradation during prolonged heating [15,16]. Similar trends have been reported for other leafy vegetables, including spinach, amaranth, and kale, where cooking methods involving direct contact with water resulted in lower apparent amino acid retention than steaming or microwave processing [17,18].

Stir-frying produced intermediate results. Although amino acid concentrations were generally lower than those recorded after steaming and microwaving, apparent retention remained higher than in boiled samples. The shorter processing time and absence of an aqueous medium may limit amino acid losses despite exposure to elevated temperatures [19,26,27].

Although cysteine and methionine exhibited the greatest apparent reductions after cooking, these results should be interpreted with caution because sulfur-containing amino acids are susceptible to degradation during conventional acid hydrolysis in the absence of prior performic acid oxidation. Cysteine content decreased from 1.95 mg/g DW in raw leaves to 1.31 mg/g DW after boiling, corresponding to a reduction of approximately 33%, whereas methionine declined by nearly 19%. The pronounced susceptibility of sulfur-containing amino acids to thermal degradation is well documented and has been attributed to the high reactivity of sulfhydryl and sulfur-containing functional groups, which are readily oxidized during heating [28,29,30].

Among the evaluated cooking methods, steaming was the most effective in preserving the amino acid profile, followed closely by microwave treatment. In contrast, boiling caused the greatest reductions, most likely due to the combined effects of thermal degradation and leaching into the cooking medium. Evaluation of amino acid quality indices demonstrated that culinary processing had only a limited effect on the relative distribution of essential and non-essential amino acids. The EAA/NEAA ratio remained remarkably stable across all treatments, ranging from 0.698 to 0.705, while the contribution of essential amino acids to total amino acids (EAA/TAA) remained close to 41% in all samples. Taken together, these findings indicate that thermal processing reduces absolute amino acid concentrations without substantially altering the overall amino acid balance. Steamed leaves exhibited the highest apparent retention of both ΣEAA and ΣNEAA on a dry-weight basis, suggesting that limited contact with water better preserves the amino acid profile than conventional boiling. The noted EAA/TAA values exceed the 40% threshold frequently considered indicative of good-quality dietary protein, suggesting that *C. maxima* leaves may serve as a valuable complementary protein source in plant-based diets.

### 2.3. Individual Phenolic Acids and Flavonoids

The concentrations of individual phenolic acids and flavonoids in raw and processed *C. maxima* leaves are presented in Table 3. Significant differences (*p* < 0.05) were observed among processing methods for all analyzed compounds, indicating that thermal treatment markedly influenced the phytochemical profile of pumpkin leaves. Taken together, steaming and microwaving preserved phenolic constituents more effectively than boiling and stir-frying, whereas boiling generally resulted in the greatest reductions. Among the phenolic acids, caffeic acid was the predominant compound in all samples, ranging from 33.62 mg/100 g DW in boiled leaves to 75.33 mg/100 g DW in raw leaves. Ferulic and sinapic acids were also present at relatively high concentrations, suggesting that hydroxycinnamic acids constitute a major fraction of the phenolic profile of pumpkin leaves. Similar phenolic patterns have been reported in pumpkin leaves and other members of the *Cucurbitaceae* family, where caffeic, ferulic, chlorogenic, and related hydroxycinnamic acids contribute substantially to antioxidant capacity and plant defense mechanisms [10,31].

The high apparent retention noted after steaming is likely attributable to the absence of direct contact with cooking water, which minimizes leaching and oxidative degradation of phenolic compounds, in agreement with previous studies [15,16]. Microwave treatment also resulted in relatively high apparent retention of phenolic acids. For example, chlorogenic acid, caffeic acid, and ferulic acid remained at 98.4%, 97.1%, and 97.8% of their original concentrations. The favorable performance of microwaving may be explained by the shorter processing time and reduced exposure to oxygen compared with conventional cooking methods [21].

Stir-frying produced intermediate apparent retention of phenolic acids. Nevertheless, concentrations of caffeic acid (34.51 mg/100 g DW), ferulic acid (22.73 mg/100 g DW), and sinapic acid (15.34 mg/100 g DW) remained significantly lower than those detected after steaming and microwaving. The elevated processing temperature probably accelerated oxidation and thermal degradation despite the absence of direct contact with water [10,16]. A similar trend was observed for flavonoids. Rutin was the predominant flavonoid in pumpkin leaves, followed by kaempferol and quercetin. Raw leaves contained 28.15 mg/100 g DW of rutin and 16.17 mg/100 g DW of kaempferol, indicating that flavonol derivatives constitute a major class of bioactive compounds in this vegetable [32,33,34,35,36,37,38].

Limited contact with water and mild thermal conditions probably reduced leaching and hydrolysis of flavonoid glycosides [16]. Microwaving also maintained high concentrations of flavonoids, particularly rutin, quercetin, and astragalin. For several compounds, rutin concentrations remained above 93% of the raw sample. These findings support previous reports indicating that microwave processing can effectively preserve flavonoid content by minimizing both thermal exposure and leaching losses [21].

Boiling resulted in the greatest flavonoid losses. Isoquercitrin and astragalin concentrations decreased by nearly 49% and 41% compared with raw leaves. The decline was also evident for rutin, kaempferol, and myricetin. Such reductions are commonly attributed to the water solubility of flavonoid glycosides and their transfer into cooking water during prolonged heating [15].

The mechanism underlying this observation remains unclear and warrants further investigation [17]. Overall, steaming preserved phenolic acids and flavonoids more effectively than the other cooking methods. In contrast, boiling caused the greatest losses due to leaching and thermal degradation, while stir-frying resulted in intermediate apparent retention but substantial reductions in several hydroxycinnamic acids. These findings suggest that cooking methods involving limited water contact are more effective for preserving the phytochemical quality of *C. maxima* leaves and may better preserve phytochemicals associated with health-promoting properties. Correlation analysis revealed strong positive relationships between total phenolic content and FRAP, supporting the contribution of phenolic constituents to the antioxidant capacity of pumpkin leaves. Similar associations have been reported for other leafy vegetables, in which caffeic acid, ferulic acid and flavonoid glycosides are major determinants of reducing power and radical scavenging activity.

The HPLC-DAD method was validated prior to quantitative analysis, and the validation parameters are presented in Table 4. Excellent linearity was obtained for all analytes (R^2^ > 0.999), while LOD and LOQ values indicated high analytical sensitivity. Precision (RSD ≤ 3.8%) and recovery (95.2–101.3%) demonstrated good repeatability and accuracy of the method, confirming its suitability for the quantitative determination of phenolic compounds in pumpkin leaves.

### 2.4. Total Phenolic Content, Total Flavonoid Content, Carotenoids and Antioxidant Activity

The effects of different culinary processing methods on total phenolic content (TPC), total flavonoid content (TFC), total carotenoids, and antioxidant activity of *C. maxima* leaves are presented in Table 5. Significant differences (*p* < 0.05) were detected among treatments for all analyzed parameters, indicating that thermal processing substantially influenced both phytochemical composition and antioxidant potential.

Raw pumpkin leaves contained high concentrations of bioactive compounds, including 482.3 mg GAE/100 g DW of total phenolics, 177.1 mg QE/100 g DW of total flavonoids, and 12.8 mg/100 g DW of total carotenoids. However, the response of these compounds to culinary processing varied considerably depending on the cooking method applied.

Steaming was the most effective treatment for preserving phytochemicals. The TPC of steamed leaves (476.0 mg GAE/100 g DW) remained statistically comparable to that of raw leaves, representing approximately 98.7% apparent retention of total phenolics. Similarly, steamed samples retained 97.1% of total flavonoids and 95.3% of carotenoids. Thermal disruption of chloroplast ultrastructure may initially increase carotenoid extractability by releasing pigments from intracellular structures. However, prolonged heating accelerates trans–cis isomerization and oxidative cleavage, resulting in the degradation of native carotenoids and lower measurable concentrations. The superior apparent retention of phenolics and carotenoids after steaming is likely attributable to the absence of direct contact with cooking water, which minimizes leaching losses and limits oxidative degradation. Previous studies have consistently reported superior preservation of phytochemicals in steamed vegetables compared with boiling, particularly for phenolic acids, flavonoids, and carotenoids [15,16].

Microwave processing also maintained relatively high concentrations of bioactive compounds. Total phenolics, flavonoids, and carotenoids reached 451.6 mg GAE/100 g DW, 128.5 mg QE/100 g DW, and 10.9 mg/100 g DW. The comparatively good apparent retention of phytochemicals after microwaving has been associated with shorter heating times and reduced exposure to oxygen and water, thereby limiting degradation reactions [21]. Nevertheless, the reductions observed relative to steaming suggest that some thermal decomposition of sensitive compounds still occurred during microwave treatment.

Boiling resulted in the greatest losses of phytochemicals among all treatments. Compared with raw leaves, TPC, TFC, and carotenoid contents decreased by approximately 17.4%, 44.6%, and 35.9%. These reductions are likely associated with the migration of water-soluble compounds into the cooking medium and the degradation of thermolabile constituents. Phenolic compounds, particularly low-molecular-weight phenolic acids and flavonoid glycosides, are susceptible to leaching during boiling, while carotenoids may undergo oxidation and isomerization when exposed to heat for prolonged periods [15,17].

Stir-frying produced intermediate phytochemical apparent retention. Although TPC (425.2 mg GAE/100 g DW), TFC (102.1 mg QE/100 g DW), and carotenoid concentrations (9.4 mg/100 g DW) were significantly lower than those recorded for steamed leaves, their values remained higher than those detected after boiling. The elevated temperatures associated with stir-frying may accelerate oxidation and degradation reactions; however, the shorter processing time and limited contact with water likely reduce phytochemical losses compared with boiling [16].

The antioxidant activity results generally followed the same pattern as phytochemical apparent retention. Steamed leaves exhibited the highest ferric reducing antioxidant power (FRAP), reaching 4.12 mmol Fe^2+^/100 g DW, significantly exceeding the value recorded for raw leaves (3.54 mmol Fe^2+^/100 g DW). Notably, despite only minor reductions in TPC and TFC relative to raw leaves, antioxidant activity increased after steaming. This observation may be explained by heat-induced disruption of plant tissues, which can enhance the extractability and accessibility of antioxidant compounds without necessarily increasing their total concentration. Consequently, the higher antioxidant activity measured after steaming may reflect improved extractability rather than an increase in the total amount of bioactive compounds.

Microwaved samples also exhibited high antioxidant activity compared with raw leaves, with a FRAP value of 3.88 mmol Fe^2+^/100 g DW and high DPPH and ABTS antioxidant capacities, expressed as Trolox equivalents. These results further support the hypothesis that moderate thermal processing may improve the availability of antioxidant compounds while minimizing their degradation.

In contrast, boiling produced the lowest antioxidant activity among all treatments. The FRAP value decreased to 2.78 mmol Fe^2+^/100 g DW, while DPPH and ABTS antioxidant capacities also decreased, indicating reduced radical scavenging capacity. The marked reduction in antioxidant activity corresponded closely with the pronounced decreases in total phenolic content, total flavonoid content, and carotenoids, indicating that losses of these bioactive compounds substantially contributed to the diminished antioxidant capacity of boiled leaves.

Stir-fried leaves displayed intermediate antioxidant activity. Although TPC, TFC, and carotenoid contents were lower than those of steamed samples, the FRAP value remained relatively high (3.42 mmol Fe^2+^/100 g DW). However, stir-frying resulted in lower DPPH and ABTS antioxidant capacities than those measured in raw leaves, indicating a moderate reduction in radical scavenging activity following stir-frying. These findings suggest that antioxidant capacity depends not only on the total concentration of phytochemicals but also on their chemical composition, extractability, and interactions within the plant matrix. Taken together, the present results demonstrate a generally consistent relationship between phytochemical apparent retention and antioxidant activity across the evaluated cooking methods.

From a practical nutritional perspective, the measured differences among cooking methods may be relevant for habitual dietary intake. Under the experimental conditions used in this study, steaming showed the highest apparent retention of most nutrients and phytochemicals. Microwave treatment also provided good apparent retention of bioactive compounds, whereas boiling resulted in the greatest losses. Overall, steaming proved to be the most effective cooking method for preserving both phytochemical composition and antioxidant properties, followed by microwave processing, whereas boiling caused the greatest deterioration.

### 2.5. Inhibitory Activity Against α-Amylase

The α-amylase inhibitory activity of *C. maxima* leaf extracts increased in a concentration-dependent manner over the tested concentration range (0.05–0.20 mg/mL) (Table 6). At all concentrations, steamed extracts exhibited the highest inhibitory activity among the culinary treatments, followed by microwaved, raw, stir-fried and boiled samples.

At the highest concentration (0.20 mg/mL), steamed extracts inhibited α-amylase by 57.86%, whereas boiled samples exhibited the lowest inhibition (41.87%). Raw and microwaved extracts showed intermediate inhibitory activities of 54.83% and 51.74%, respectively.

As expected, acarbose exhibited significantly stronger inhibition than all plant extracts, reaching 92.36% at 0.20 mg/mL.

These results demonstrate that culinary processing markedly influenced α-amylase inhibitory activity, with steaming providing the greatest preservation of enzyme inhibitory potential, whereas boiling resulted in the largest reduction.

The stronger α-amylase inhibitory activity found in steamed and microwaved samples generally paralleled the higher apparent retention of phenolic compounds and flavonoids, suggesting that these phytochemicals may contribute to the inhibition of α-amylase. However, the present study was not designed to establish direct relationships between individual compounds and α-amylase inhibitory activity [39].

### 2.6. Effect of Culinary Processing on Antinutritional Compounds

The concentrations of major antinutritional compounds, including phytates, oxalates, and tannins, in raw and processed *C. maxima* leaves are presented in Table 7. Significant differences (*p* < 0.05) were observed among processing methods for all analyzed compounds, demonstrating that culinary treatment substantially affected the antinutritional profile of pumpkin leaves. In general, all processing methods reduced antinutritional factors relative to the raw material, although the magnitude of reduction varied considerably depending on the cooking technique employed.

Raw pumpkin leaves contained relatively high concentrations of phytates (81.05 mg/g), oxalates (85.46 mg/g), and tannins (42.34 mg/g). These compounds are naturally present in many leafy vegetables and serve important physiological functions in plants; however, they may reduce nutrient bioavailability in humans through the formation of insoluble complexes with minerals, proteins, and digestive enzymes [40,41]. Consequently, reducing their concentration through appropriate processing may improve the nutritional quality and may reduce the mineral-binding potential of leafy vegetables.

Among all treatments, boiling was the most effective method for reducing antinutritional compounds. Phytate concentration decreased from 81.05 to 16.62 mg/g, corresponding to an apparent retention of only 20.5% relative to raw leaves. Similarly, oxalate concentration declined by approximately 82%, while tannins were reduced by nearly 50%. The present study demonstrated that antinutritional compounds were removed during boiling. The marked reduction is most likely attributable to the water solubility of these compounds and their diffusion into the cooking medium. Previous studies have shown that boiling is particularly effective in reducing phytates, oxalates, and tannins because these compounds can readily leach into cooking water during thermal processing [42].

The reduction in oxalates found after boiling is of particular nutritional significance. Oxalates can bind calcium and other divalent minerals, thereby decreasing mineral absorption and potentially contributing to kidney stone formation in susceptible individuals. The decline from 85.46 to 15.56 mg/g suggests that boiling may substantially improve the mineral bioavailability of pumpkin leaves. Similar reductions in oxalate content following boiling have been reported for spinach, amaranth, and other green leafy vegetables [43].

Microwaving also reduced antinutritional compounds, although to a lesser extent than boiling. Apparent retention values of 42.7% for phytates, 41.8% for oxalates, and 69.3% for tannins indicate moderate reductions following microwave treatment. The effectiveness of microwaving likely results from thermal degradation of heat-sensitive compounds combined with limited moisture-mediated losses. However, because microwave processing generally involves minimal water, the removal of soluble anti-nutrients is less extensive than during boiling. Similar observations have been reported in studies comparing microwave and conventional cooking methods for leafy vegetables [21].

Steaming preserved the highest concentrations of antinutritional compounds among all processing methods. Apparent retention values reached 80.6% for phytates, 75.4% for oxalates, and 79.0% for tannins. These results are consistent with the limited contact between plant tissues and water during steaming. Although steaming effectively preserves desirable nutrients and phytochemicals, it appears less effective in removing antinutritional factors. This finding highlights the nutritional trade-off frequently noted in vegetable processing, where methods that maximize apparent retention of beneficial compounds may simultaneously preserve compounds that limit nutrient bioavailability [15].

Stir-frying produced intermediate reductions in antinutritional compounds. Phytate apparent retention decreased to 54.7%, while oxalate and tannin apparent retention reached 34.6% and 72.3%. The substantial reduction in oxalates observed after stir-frying may be associated with thermal degradation and structural changes occurring at elevated temperatures. In contrast, the comparatively higher apparent retention of tannins suggests that these compounds are more resistant to degradation under dry-heat conditions than phytates and oxalates. Similar patterns have been noted in thermally processed vegetables and legumes, where different antinutritional compounds exhibit distinct responses to heat treatment depending on their chemical structure and stability [42,44].

The results indicate that phytates and oxalates were generally more susceptible to processing-induced reductions than tannins. This observation may be explained by differences in chemical properties. Phytates and soluble oxalates readily dissolve in water and are therefore particularly vulnerable to leaching during boiling, whereas tannins often form complexes with proteins and cell wall components, which may reduce their mobility and increase their thermal stability [42].

When the antinutritional results are considered together with the phytochemical and antioxidant findings presented earlier, an important balance emerges. Steaming was identified as the most effective method for preserving phenolic compounds, flavonoids, carotenoids, and antioxidant activity, yet it also retained the highest levels of antinutritional factors. Conversely, boiling achieved the greatest reduction in phytates, oxalates, and tannins but resulted in substantial losses of beneficial phytochemicals. Microwave processing appears to provide a compromise between these two extremes by maintaining relatively high levels of bioactive compounds while simultaneously reducing antinutritional constituents to a moderate extent.

From a nutritional perspective, the choice of processing method therefore depends on the desired outcome. If the primary objective is to maximize phytochemical apparent retention and antioxidant capacity, steaming appears to be the most suitable technique. However, if reduction in anti-nutritional compounds and improvement of mineral bioavailability are prioritized, boiling may offer greater benefits. Microwaving may represent the most balanced approach, providing acceptable apparent retention of health-promoting phytochemicals while substantially lowering antinutritional factors.

Overall, these findings suggest that culinary processing significantly modifies the antinutritional profile of *C. maxima* leaves. The substantial reductions in phytates, oxalates, and tannins noted after boiling and microwaving suggest that appropriate household processing can improve the nutritional value and potential mineral availability of pumpkin leaves while maintaining a considerable proportion of their bioactive constituents.

Although boiling produced the greatest reduction in antinutritional compounds, it simultaneously caused the highest losses of nutrients and phytochemicals. Therefore, the selection of processing methods should balance antinutrient reduction with preservation of nutritional quality.

The reduction in antinutritional compounds during boiling is primarily attributed to their high water solubility and diffusion into the cooking medium, whereas thermal degradation and hydrolysis may additionally contribute depending on the chemical nature of individual compounds.

### 2.7. Heatmap and Hierarchical Cluster Analysis of the Effects of Culinary Processing on Pumpkin Leaves

A heatmap combined with hierarchical cluster analysis (HCA) was used to visualize similarities among the raw and processed *C. maxima* leaf samples and to explore relationships among the measured variables. The analysis included nutritional parameters, phenolic compounds, antioxidant activity, α-amylase inhibitory activity, and antinutritional factors.

Prior to clustering, all variables were standardized using Z-score normalization (mean = 0, standard deviation = 1) to eliminate differences in measurement scales and ensure equal contribution of each variable to the analysis. Euclidean distance was used as the similarity measure, and hierarchical clustering was performed using Ward’s minimum variance linkage method. Both samples and variables were clustered simultaneously, and the results were visualized as a heatmap accompanied by dendrograms.

In the heatmap, warmer colors (yellow to red) indicate relatively higher standardized values, whereas cooler colors (light blue to dark blue) represent relatively lower standardized values. The heatmap and dendrograms were generated using OriginPro 2025 (OriginLab Corporation, Northampton, MA, USA).

### 2.8. Study Limitations

**Figure 1:**
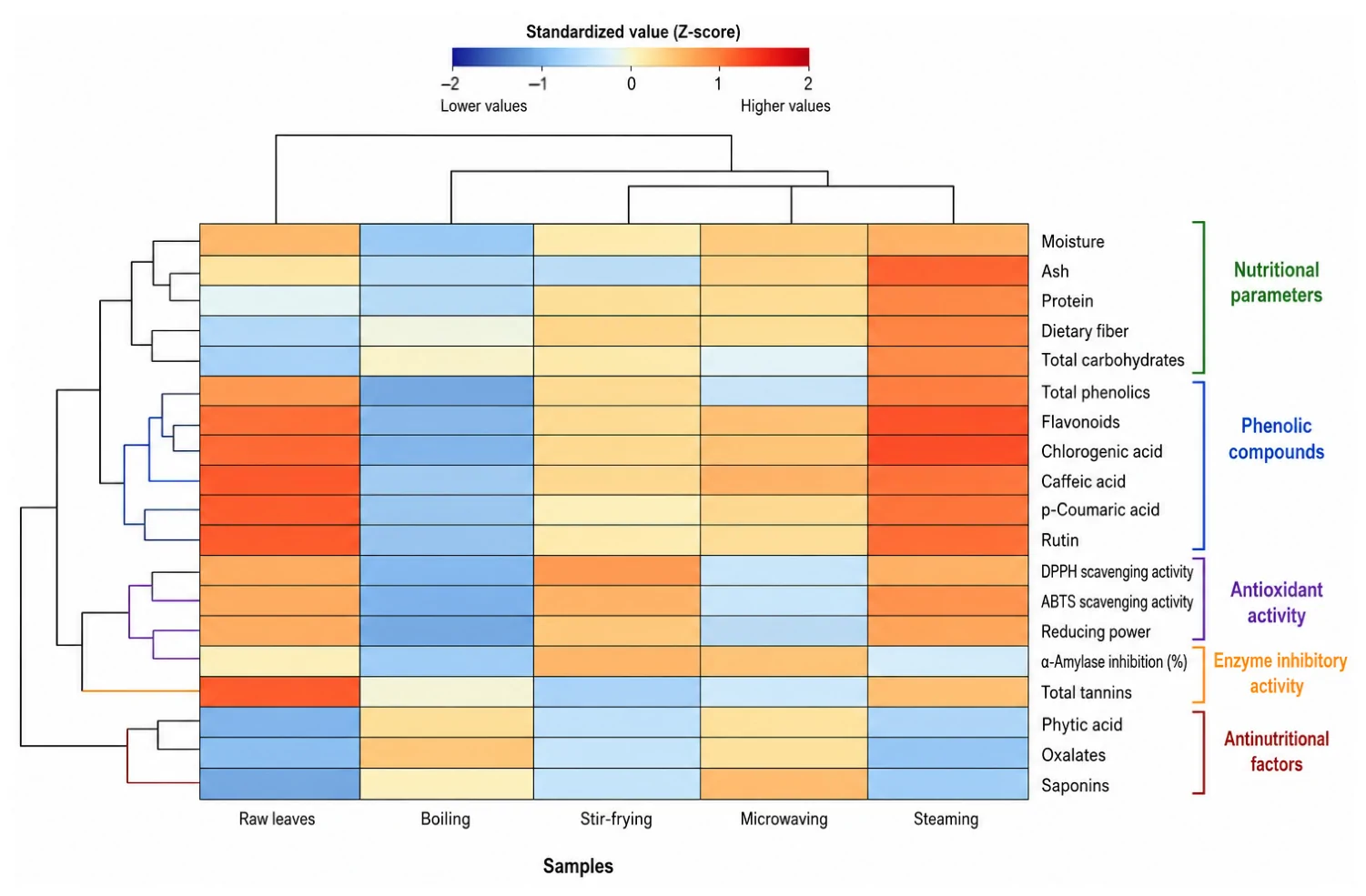
Heatmap and hierarchical cluster analysis (HCA) showing the relationships among nutritional parameters, phenolic compounds, antioxidant activity, enzyme inhibitory properties, and antinutritional factors in raw and processed *C. maxima* leaves. Samples and variables were clustered using Euclidean distance and Ward’s linkage method. Higher standardized values are represented by warmer colors, whereas lower values are represented by cooler colors.

The present study has several limitations that should be considered when interpreting the results (Figure 1). First, the functional properties of pumpkin leaf extracts, including antioxidant capacity and α-amylase activities, were evaluated exclusively using in vitro assays. Although these methods provide valuable preliminary information, they do not fully reflect the complex physiological conditions of the human gastrointestinal tract and therefore cannot be directly translated into biological or clinical effects.

Second, the investigation was performed using a single cultivar of *C. maxima* harvested at one developmental stage. The nutritional composition, phytochemical profile, and response to thermal processing may vary depending on genotype, environmental conditions, agronomic practices, harvest maturity, and postharvest storage.

Third, only four standardized household cooking methods were investigated under fixed processing conditions. Domestic cooking practices differ considerably in terms of temperature, cooking time, water-to-vegetable ratio, equipment, and preparation techniques, which may influence nutrient relative retention and phytochemical stability.

Furthermore, no simulated gastrointestinal digestion, cellular experiments, animal studies, or human intervention studies were conducted. Consequently, the bioaccessibility, bioavailability, metabolism, and physiological activity of the retained bioactive compounds remain to be established.

Future research should therefore include multiple cultivars, different maturity stages, in vitro gastrointestinal digestion models, cell culture experiments, and in vivo studies to verify the biological relevance and nutritional implications of the present findings.

## 3. Materials and Methods

### 3.1. Plant Material

Fresh leaves of *Cucurbita maxima* L. ‘Amazonka’ were collected during the intensive vegetative growth stage in 2025 from a cultivated field located in the Lublin region (51°15′ N, 22°34′ E), Poland. The plants were randomly divided into three biological replicates, each consisting of leaves collected from five individual plants. Plants were randomly selected across the experimental field to minimize positional effects. Leaves within each biological replicate were pooled, thoroughly homogenized, and used as one representative biological sample for all subsequent analyses. Each pooled biological replicate was homogenized and divided into five portions corresponding to the experimental treatments (raw, boiling, steaming, microwaving, and stir-frying). Each cooking treatment was applied independently to every biological replicate.

Only healthy, undamaged, and fully expanded leaves were selected for the study. The collected material was transported to the laboratory under refrigerated conditions (4 °C) and processed within 24 h of collection. Before analysis, the leaves were manually cleaned to remove contaminants and thoroughly rinsed with distilled water. Excess surface moisture was removed using laboratory filter paper. The prepared plant material was then divided into portions corresponding to different culinary processing methods.

For each treatment, three independent biological replicates (each representing pooled leaves from five plants) were analyzed. All analytical determinations were performed in triplicate.

### 3.2. Culinary Processing

The cooking conditions were selected to simulate typical household culinary practices commonly used for leafy vegetables while ensuring reproducible processing conditions. The applied temperatures, cooking times, microwave power, and stir-frying conditions were based on previously published studies evaluating the effects of domestic cooking on vegetable quality and were selected to achieve complete cooking without excessive thermal degradation.

Pumpkin leaves were subjected to four culinary processing methods: boiling, steaming, microwaving, and stir-frying. Untreated fresh leaves were used as the control sample. Cooking times were selected on the basis of preliminary trials to obtain acceptable culinary softness while avoiding excessive tissue disintegration.

For boiling treatment, 100 g of leaves were immersed in 150 mL of distilled water and heated at approximately 95 °C for 10 min in a covered stainless-steel vessel. Steaming was performed using a stainless-steel steaming system containing boiling water (98 °C). Leaf samples (100 g) were exposed to steam for 10 min and subsequently cooled under chilled conditions.

Microwave processing was carried out using a domestic microwave oven (Model 775002V01, Stalgast, Warsaw, Poland) operating at 2450 MHz. Approximately 100 g of pumpkin leaves were placed in a glass container with 12 mL of distilled water and heated at 700 W for 3 min. Following treatment, samples were rapidly cooled under chilled conditions before further processing.

For stir-frying, 10 mL of extra virgin olive oil was preheated in a frying pan (Gerlach, Drzewica, Poland) before adding 100 g of pumpkin leaves. The material was stir-fried for 2 min at approximately 150 °C with continuous mixing. The use of extra virgin olive oil may have contributed to the measured lipid content and may also have influenced the antioxidant activity of the stir-fried samples. Temperature during thermal treatment was monitored using a digital food thermometer (Browin, Lodz, Poland). Following culinary processing, all samples were immediately frozen, lyophilized using a freeze dryer (Alpha 1–2 LD Plus, Martin Christ, Osterode am Harz, Germany), ground into powder, and stored at −20 °C in airtight aluminum containers until further analysis.

Each culinary treatment was applied independently to the three biological replicates described in Section 3.1. Cooking water, steaming condensate and frying oil were discarded after processing and were not included in chemical analyses. Therefore, the present study evaluates the composition of the cooked leaves only.

### 3.3. Proximate Composition Analysis

Moisture was determined according to AOAC 925.10 (2022) [44] by drying samples in a laboratory oven at 105 °C until no further change in weight was observed. The mineral residue (ash) was quantified using AOAC 942.05 (2023) [45]. For this purpose, samples were combusted in a muffle furnace at 550 °C until complete mineralization, indicated by the formation of white or pale gray ash.

The protein fraction was assessed using the Kjeldahl procedure [46]. Nitrogen values obtained from the analysis were multiplied by a factor of 6.25 to estimate total protein content.

Lipid content was measured by Soxhlet extraction [47] using petroleum ether as the extracting medium.

Total dietary fiber (TDF) was determined according to AOAC Official Method 991.43 [48,49] using the enzymatic–gravimetric method. Samples were sequentially digested with heat-stable α-amylase, protease and amyloglucosidase to remove digestible starch and protein. Insoluble dietary fiber was recovered by filtration, whereas soluble dietary fiber was precipitated with 95% ethanol, filtered, dried, and corrected for residual protein and ash, and the sum of both fractions was expressed as total dietary fiber (g/100 g dry weight). Available carbohydrates were calculated by difference after subtraction of moisture, ash, protein, fat and total dietary fiber from the total sample weight. Available carbohydrate content was expressed as g/100 g sample. All analyses were performed in triplicate, and the results are expressed on both fresh-weight (FW) and dry-weight (DW) bases.

### 3.4. Amino Acid Analysis

Approximately 0.2 g of freeze-dried sample was accurately weighed for analysis. To enable the determination of sulfur-containing amino acids (methionine and cysteine), separate sample portions were subjected to pre-oxidation with freshly prepared performic acid under refrigerated conditions (0–4 °C) for 16 h according to the procedure described by Moore (1963) [50]. The oxidation reaction was terminated by the addition of sodium metabisulfite prior to acid hydrolysis.

Subsequently, samples were hydrolyzed with 6 M hydrochloric acid in sealed hydrolysis ampoules under a nitrogen atmosphere at 110 °C for 24 h to prevent oxidative degradation of amino acids during hydrolysis. Following hydrolysis, the solutions were filtered, evaporated under reduced pressure to remove residual hydrochloric acid, and diluted with sodium citrate buffer (pH 2.2).

Amino acids were separated and quantified using an automatic amino acid analyzer (S433D, Sykam GmbH, Eresing, Germany) equipped with post-column ninhydrin derivatization. Individual amino acids were identified by comparison of retention times with certified amino acid standards and quantified using external calibration. Tryptophan was not determined because it is degraded during acid hydrolysis.

Essential amino acids (EAA), non-essential amino acids (NEAA), total amino acids (TAA), and the EAA/TAA ratio were calculated from the analytical data.

The concentrations of individual amino acids were expressed as mg/gDW. All analyses were conducted in triplicate.

### 3.5. Extraction and HPLC-DAD Analysis of Phenolic Compounds

Phenolic compounds were extracted from freeze-dried pumpkin leaves using a modified procedure based on previously described methods. Briefly, samples were homogenized for 2 min using an Ultra-Turrax homogenizer (IKA-Werke GmbH & Co. KG, Staufen, Germany) with 2 mL of 80% acidified methanol (*v*/*v*, containing hydrochloric acid). The homogenates were centrifuged at 10,000 rpm for 15 min at 4 °C, and the supernatants were filtered through Whatman No. 1 filter paper prior to analysis.

HPLC-DAD analysis was performed using a Shimadzu LC-20A system (Shimadzu Corporation, Kyoto, Japan) equipped with a quaternary pump, autosampler, column oven, and diode-array detector. Separation was achieved on a reversed-phase C18 column (Phenomenex Luna C18, 250 × 4.6 mm, 5 μm) maintained at 30 °C.

The mobile phase consisted of solvent A (0.1% formic acid in ultrapure water, *v*/*v*) and solvent B (acetonitrile). The gradient program was as follows: 0–5 min, 5% B; 5–20 min, 5–20% B; 20–35 min, 20–40% B; 35–40 min, 40–60% B; 40–45 min, 60% B, followed by re-equilibration for 10 min. The flow rate was 1.0 mL/min, and the injection volume was 20 μL.

Phenolic compounds were monitored at 280 nm (gallic acid, protocatechuic acid, and vanillic acid), 320 nm (chlorogenic acid, caffeic acid, p-coumaric acid, ferulic acid, and sinapic acid), and 360 nm (flavonoids: rutin, quercetin, isoquercitrin, astragalin, kaempferol, and myricetin).

Identification of compounds was carried out by comparing apparent retention times and UV–Vis spectra with those of authentic analytical standards. Quantification was performed using external calibration curves prepared at six concentration levels (1–100 mg L^−1^) using reference standards (Sigma-Aldrich, St. Louis, MO, USA).

Calibration curves showed good linearity (R^2^ > 0.999). Limits of detection (LOD) and quantification (LOQ) were calculated based on signal-to-noise ratios of 3 and 10, respectively. Method validation included evaluation of intra- and inter-day precision (expressed as %RSD) and recovery (spiked samples).

The concentrations of individual phenolic compounds were expressed as mg/100 g dry matter (DM).

### 3.6. Determination of Total Phenolic Content (TPC)

Extracts for the determination of total phenolic content (TPC), total flavonoid content (TFC), and antioxidant activity were prepared from lyophilized pumpkin leaf samples. Briefly, 0.75 g of freeze-dried leaf material was homogenized in a mortar with 25 mL of 80% methanol (Sigma-Aldrich, St. Louis, MO, USA), which was added gradually during the extraction process. Extraction was carried out for 4 h under dark conditions at 22 °C. Following extraction, the mixtures were filtered through a sintered glass funnel into 25 mL volumetric flasks. Three independent extracts were prepared from each biological replicate.

The obtained extracts were stored at −25 °C in the absence of light until analysis.

The total phenolic content was quantified using the Folin–Ciocalteu [50] colorimetric method. An aliquot (0.5 mL) of the methanolic pumpkin leaf extracts was combined with a tenfold diluted Folin–Ciocalteu reagent and 7.5% sodium carbonate solution. The reaction mixtures were incubated in the dark for 30 min, after which their absorbance was recorded at 760 nm using a UV-2600i Plus spectrophotometer (Shimadzu, Kyoto, Japan). Phenolic compounds were quantified using a gallic acid calibration curve, and the results were expressed as mg GAE/100 g DW.

### 3.7. Determination of Total Flavonoid Content (TFC)

The total flavonoid content (TFC) was determined using a colorimetric assay. Briefly, 0.5 mL of the methanolic extract prepared as described in Section 3.6 was mixed with 2.0 mL of distilled water and 150 μL of 5% sodium nitrite (NaNO_2_) solution. The mixture was allowed to stand in the dark for 5 min. Subsequently, 150 μL of 10% aluminum chloride (AlCl_3_) solution was added, followed by a further dark incubation period of 6 min. Thereafter, 1.0 mL of 1 M sodium hydroxide (NaOH) and 1.0 mL of distilled water were introduced into the reaction mixture. The absorbance of the resulting solution was measured at 510 nm using a UV-2600i Plus spectrophotometer (Shimadzu, Kyoto, Japan) [51].

Quantification of flavonoids was performed using a calibration curve prepared with quercetin as the reference standard. The results were expressed as mg QE/100 g DW.

### 3.8. Determination of Total Carotenoid

Total carotenoid content was determined according to the spectrophotometric method described by Nagata and Yamashita [52]. Freeze-dried pumpkin leaf samples (0.75 g) were homogenized with 25 mL of an acetone/hexane mixture (4:6, *v*/*v*) until the plant material became colorless, indicating complete pigment extraction. The homogenates were filtered through Whatman No. 1 filter paper, and the absorbance of the clear extracts was measured at 453, 505, 645, and 663 nm using a UV-2600i Plus spectrophotometer (Shimadzu, Kyoto, Japan).

Total carotenoid content was calculated using the equation proposed by Nagata and Yamashita [52]:

Carotenoids (mg/100 mL) = 0.216 × A663 − 1.22 × A645 − 0.304 × A505 + 0.452 × A453

The results were converted to mg/100 g dry weight (DW). All analyses were performed in triplicate.

### 3.9. Antioxidant Activity Assays

#### 3.9.1. DPPH Radical Scavenging Activity

The DPPH radical scavenging activity was determined according to the method of Brand-Williams et al. (1995) [52] with minor modifications. A freshly prepared methanolic solution of DPPH (0.1 mM) was used for all measurements. Pumpkin leaf extracts were prepared as described in Section 3.6 and serially diluted to obtain concentrations ranging from 50 to 2000 μg mL^−1^.

An aliquot of 100 µL of the extract solution was mixed with 3.9 mL of freshly prepared 0.1 mM DPPH solution.

The reaction mixtures were vortexed and incubated in the dark at room temperature for 30 min. The absorbance was then measured at 517 nm using a UV-2600i Plus spectrophotometer (Shimadzu, Kyoto, Japan). A control containing methanol instead of the extract was prepared under identical conditions.

The antioxidant capacity was determined using the DPPH radical scavenging assay and expressed as milligrams of Trolox equivalents (mg TE) per 100 g dry matter (DW) based on a Trolox calibration curve.

All analyses were performed using three independent biological replicates.

#### 3.9.2. ABTS Radical Cation Decolorization Assay

The ABTS radical scavenging activity was determined according to the method of Re et al. (1999) [53] with minor modifications. The ABTS radical cation (ABTS•^+^) was generated by mixing equal volumes of 14 mM ABTS solution and 4.9 mM potassium persulfate solution, followed by incubation in the dark at room temperature for 12–16 h. Prior to analysis, the resulting ABTS solution was diluted with distilled water to obtain an absorbance of 0.70 ± 0.02 at 734 nm.

Pumpkin leaf extracts were prepared as described in Section 3.6 and tested at concentrations ranging from 50 to 2000 μg/mL. For each determination, 40 μL of the extract was mixed with 4.0 mL of the ABTS working solution, vortexed, and incubated in the dark at room temperature for 6 min. The absorbance was then measured at 734 nm using a UV-2600i Plus spectrophotometer (Shimadzu, Kyoto, Japan). A control containing methanol instead of the extract was prepared under identical conditions.

The antioxidant capacity was determined using the ABTS assay and expressed as milligrams of Trolox equivalents (mg TE) per 100 g dry matter (DM). Trolox was used as the calibration standard.

#### 3.9.3. Ferric Reducing Antioxidant Power (FRAP)

Ferric reducing antioxidant power (FRAP) was determined according to the method of Benzie and Strain (1996) [54] with minor modifications. The FRAP reagent consisted of 300 mM sodium acetate buffer (pH 3.6), 10 mM TPTZ solution, and 20 mM ferric chloride solution mixed in the appropriate proportions. An aliquot of the extract (10 μL) was mixed with freshly prepared FRAP reagent. The reaction was monitored after 30 min incubation at 37 °C at 593 nm using a UV-Vis spectrophotometer (Shimadzu, Kyoto, Japan). A calibration curve was prepared using freshly prepared ferrous sulfate (FeSO_4_·7H_2_O) standard solutions, and the results were expressed as mmol Fe^2+^ equivalents per 100 g DW.

### 3.10. α-Amylase Inhibitory Activity

The inhibitory activity against α-amylase was determined according to the method described by Subramanian et al. [55] with minor modifications concerning the reaction volume and extract concentrations. Leaf extracts and acarbose (positive control) were dissolved in 20 mM sodium phosphate buffer (pH 6.9) containing 0.006 M sodium chloride to obtain final concentrations of 0.05, 0.10, 0.15, and 0.20 mg/mL.

For each assay, 40 μL of the extract or acarbose solution was mixed with 200 μL of α-amylase solution (1.0 U/mL) prepared in the same buffer and pre-incubated at 25 °C for 30 min. Fresh enzyme solution (1.0 U/mL) was prepared immediately before each experiment using sodium phosphate buffer.

The enzymatic reaction was initiated by adding 400 μL of 0.25% (*w*/*v*) soluble starch prepared in sodium phosphate buffer (pH 6.9), followed by incubation at 37 °C for 5 min.

The reaction was terminated by adding 1.0 mL of 3,5-dinitrosalicylic acid (DNS) reagent containing 1% DNS and 12% sodium potassium tartrate in 0.4 M NaOH. The reaction mixtures were heated in a boiling water bath for 10 min, cooled to room temperature, diluted with 10 mL of distilled water, and the absorbance was measured at 540 nm using a UV-2600i Plus spectrophotometer (Shimadzu, Kyoto, Japan).

A control containing buffer instead of the extract represented 100% enzyme activity, whereas the corresponding blank was prepared by replacing the enzyme solution with buffer to correct for the intrinsic absorbance of the extracts.

The α-amylase inhibitory activity was expressed as percentage inhibition (%) according to the following equation:

% inhibition: = [(A_control_ − A_sample_)/A_control_] × 100

where A_control_ is the absorbance of the control, and A_samle_ is the absorbance of the test samples.

The concentration-dependent inhibitory activity of each extract was evaluated within the concentration range of 0.05–0.20 mg/mL, and inhibition curves were constructed by plotting percentage inhibition against extract concentration.

All measurements were carried out in triplicate for each biological replicate, and the results were expressed as mean ± standard deviation.

### 3.11. Determination of Antinutritional Compounds

Tannin, phytate, and oxalate contents were determined according to the method described by Managa et al. [56]. Tannin Content: Quick-freeze-preserved leaf samples (0.2 g) were extracted in 10 mL of 1% HCl solution. 100 µL of sample extract and 50 µL of vanillin–HCl reagent in methanol, prepared from 8% HCl in methanol and 1% vanillin in methanol, were added to the reaction mixture. Results were expressed as mg/g dry weight (DW).

Phytate Content: Phytates were extracted from freeze-dried leaf samples (0.5 g) using 100 mL of 2.4% HCl. Quantitative determination was performed using Wade’s reagent containing iron(III) chloride monohydrate and sulfosalicylic acid dissolved in distilled water. Results were expressed as mg/g dry weight (DW).

Oxalate Content: Insoluble oxalates were extracted from leaf samples (0.1 g) homogenized in 30 mL of 2 M HCl, while soluble oxalates were extracted using distilled water. CaC_2_O_4_ was then precipitated by adding 5% CaCl_2_. The resulting precipitate was separated, washed three times with 0.35 M NH_4_OH, and then dissolved in 0.5 M H_2_SO_4_. The resulting solution, containing both soluble and insoluble oxalates, was titrated with 0.1 M KMnO_4_ at 60 °C until a very faint pink color persisted for 15 min. The oxalate content was expressed as mg/g dry weight (DW).

### 3.12. Statistical Analysis

All experiments were performed using three independent biological replicates (*n* = 3). Each analytical determination was carried out in triplicate for every biological replicate, and the mean value of each biological replicate was used for statistical evaluation. Results are expressed as mean ± standard deviation (SD).

Differences among processing methods were evaluated using one-way analysis of variance (ANOVA), followed by Tukey’s honestly significant difference (HSD) post hoc test. Differences were considered statistically significant at *p* < 0.05.

Hierarchical cluster analysis (HCA) and heatmap visualization were performed using Z-score-standardized data and Ward’s hierarchical clustering method implemented in the pheatmap package. All multivariate analyses were based on the mean values obtained for each biological replicate (*n* = 3 per treatment) and were used as exploratory tools to visualize relationships among treatments and measured variables.

## 4. Conclusions

The present study extends previous research on household cooking of leafy vegetables by integrating nutritional, phytochemical, and functional analyses within a single experimental framework. This comprehensive approach provides new insight into the relationships between culinary processing, apparent nutrient retention, phenolic composition, antioxidant properties, antinutritional factors, and digestive α-amylase inhibitory activity in *C. maxima* leaves.

This study demonstrated that culinary processing significantly influences the nutritional composition, phytochemical content, antioxidant properties, α-amylase inhibitory activity, and antinutritional compounds of *C. maxima* leaves. Among the evaluated methods, steaming consistently provided the greatest apparent retention of proteins, amino acids, phenolic compounds, flavonoids, carotenoids, antioxidant capacity, and inhibitory activity against α-amylase, indicating the best overall preservation of nutritional and functional quality. Microwaving showed similar benefits and may represent an effective alternative household processing method.

In contrast, boiling resulted in the greatest reductions in amino acids, phenolic compounds, antioxidant activity, and enzyme inhibitory potential, primarily due to leaching and thermal degradation. Nevertheless, boiling was the most effective treatment for reducing antinutritional compounds such as phytates, oxalates, and tannins. Stir-frying increased lipid content and contributed to moderate apparent retention of several bioactive compounds but was generally less effective than steaming and microwaving in preserving overall nutritional quality.

The findings indicate that processing methods involving limited contact with water are most suitable for maximizing the nutritional and functional value of pumpkin leaves. Consequently, steaming appears to be the most suitable household cooking method for preserving health-promoting compounds while maintaining desirable nutritional quality. Further studies should investigate the extractability, bioavailability, and in vivo physiological effects of phytochemicals retained after processing.

However, these findings should not be interpreted as evidence of antidiabetic efficacy, since the assays were conducted exclusively in vitro.

Although steaming provided the best preservation of nutritional and functional properties under the conditions of this study, sensory characteristics such as texture, color, flavor and consumer acceptability were not evaluated. Future studies integrating nutritional quality with sensory assessment would provide more comprehensive recommendations for domestic cooking practices.

The present study evaluated only one cultivar of *C. maxima*. Since phytochemical composition may vary with genotype, environmental conditions, maturity stage and agronomic practices, caution should be exercised when extrapolating these findings to other cultivars or growing conditions.

The noted apparent retention values represent relative retention on a dry-weight basis and should therefore be interpreted within the limitations of compositional changes occurring during thermal processing.

These findings support the utilization of pumpkin leaves as a sustainable and nutritionally valuable leafy vegetable and provide evidence-based guidance for selecting household cooking methods that maximize the apparent retention of health-promoting compounds.

## Figures and Tables

**Table 1:** Effect of culinary processing on the proximate composition of *C. maxima* leaves expressed on a fresh-weight (FW) and dry-weight (DW) basis (g/100 g).

Processing Method	Moisture	Ash		Protein		Fat		Dietary Fiber		Carbohydrate	
	(%)	FW	DW	FW	DW	FW	DW	FW	DW	FW	DW
Raw leaves	86.20 ± 0.40 ^b^	2.10 ± 0.08 ^b^	15.22 ± 0.58 ^b^	4.30 ± 0.15 ^b^	31.16 ± 1.09 ^d^	0.80 ± 0.05 ^c^	5.80 ± 0.36 ^b^	3.10 ± 0.10 ^a^	22.46 ± 0.73 ^b^	3.50 ± 0.20 ^c^	25.36 ± 1.45 ^c^
Boiling	88.10 ± 0.45 ^a^	1.76 ± 0.06 ^d^	14.79 ± 0.51 ^c^	3.78 ± 0.12 ^d^	31.76 ± 1.01 ^a^	0.61 ± 0.03 ^d^	5.13 ± 0.25 ^d^	2.58 ± 0.08 ^c^	21.68 ± 0.67 ^d^	3.17 ± 0.14 ^d^	26.64 ± 1.18 ^b^
Microwaving	86.95 ± 0.38 ^b^	2.02 ± 0.05 ^c^	15.48 ± 0.39 ^a^	4.12 ± 0.13 ^c^	31.59 ± 1.00 ^b^	0.73 ± 0.04 ^c^	5.60 ± 0.31 ^c^	2.92 ± 0.07 ^b^	22.38 ± 0.54 ^c^	3.26 ± 0.16 ^cd^	24.98 ± 1.23 ^d^
Steaming	86.70 ± 0.40 ^b^	2.06 ± 0.07 ^b^	15.49 ± 0.53 ^a^	4.18 ± 0.14 ^c^	31.43 ± 1.05 ^c^	0.75 ± 0.04 ^c^	5.64 ± 0.30 ^c^	3.01 ± 0.09 ^ab^	22.63 ± 0.68 ^a^	3.30 ± 0.15 ^c^	24.81 ± 1.13 ^e^
Stir-frying	80.25 ± 0.55 ^c^	2.42 ± 0.08 ^a^	12.25 ± 0.41 ^d^	4.92 ± 0.16 ^a^	24.91 ± 0.81 ^e^	2.85 ± 0.18 ^a^	14.43 ± 0.91 ^a^	3.48 ± 0.11 ^a^	17.62 ± 0.56 ^e^	6.08 ± 0.24 ^a^	30.78 ± 1.22 ^a^

Values are expressed as mean ± standard deviation (*n* = 3). Means within a column followed by different superscript letters are significantly different (*p* < 0.05).

**Table 2:** Amino acid content (mg/g DW) in *C. maxima* pumpkin leaves before and after culinary processing.

Amino Acid	Raw Leaves	Boiling	Microwaving	Steaming	Stir-Frying
Aspartic acid	17.92 ± 0.22 ^a^	15.86 ± 0.18 ^e^	16.92 ± 0.15 ^c^	17.41 ± 0.12 ^b^	16.44 ± 0.14 ^d^
Threonine	7.18 ± 0.14 ^a^	6.22 ± 0.08 ^e^	6.84 ± 0.04 ^c^	7.02 ± 0.04 ^b^	6.51 ± 0.08 ^d^
Serine	7.62 ± 0.08 ^a^	6.55 ± 0.05 ^e^	7.18 ± 0.06 ^c^	7.47 ± 0.08 ^b^	6.83 ± 0.08 ^d^
Glutamic acid	22.52 ± 0.43 ^a^	20.64 ± 0.32 ^e^	21.72 ± 0.42 ^c^	22.14 ± 0.28 ^b^	21.16 ± 0.52 ^d^
Glycine	9.64 ± 0.14 ^a^	8.41 ± 0.18 ^e^	9.12 ± 0.14 ^c^	9.42 ± 0.18 ^b^	8.78 ± 0.17 ^d^
Alanine	14.40 ± 0.08 ^a^	12.62 ± 0.14 ^e^	13.71 ± 0.13 ^c^	14.03 ± 0.12 ^b^	13.12 ± 0.13 ^d^
Cysteine	1.95 ± 0.04 ^a^	1.31 ± 0.04 ^e^	1.62 ± 0.02 ^c^	1.81 ± 0.02 ^b^	1.49 ± 0.02 ^d^
Valine	11.75 ± 0.12 ^a^	10.21 ± 0.14 ^e^	11.11 ± 0.17 ^c^	11.53 ± 0.15 ^b^	10.74 ± 0.15 ^d^
Methionine	4.54 ± 0.14 ^a^	3.66 ± 0.12 ^e^	4.11 ± 0.14 ^c^	4.38 ± 0.14 ^b^	3.89 ± 0.12 ^d^
Isoleucine	7.56 ± 0.09 ^a^	6.64 ± 0.08 ^e^	7.16 ± 0.08 ^c^	7.41 ± 0.06 ^b^	6.91 ± 0.04 ^d^
Leucine	15.34 ± 0.11 ^a^	13.74 ± 0.18 ^e^	14.76 ± 0.12 ^c^	15.18 ± 0.12 ^b^	14.28 ± 0.13 ^d^
Tyrosine	9.64 ± 0.12 ^a^	8.61 ± 0.12 ^e^	9.22 ± 0.11 ^c^	9.52 ± 0.11 ^b^	8.95 ± 0.12 ^d^
Phenylalanine	11.59 ± 0.12 ^a^	10.23 ± 0.13 ^e^	11.02 ± 0.12 ^c^	11.41 ± 0.11 ^b^	10.67 ± 0.13 ^d^
Histidine	6.42 ± 0.04 ^a^	5.51 ± 0.10 ^e^	6.01 ± 0.08 ^c^	6.26 ± 0.06 ^b^	5.76 ± 0.04 ^d^
Lysine	13.91 ± 0.12 ^a^	12.42 ± 0.14 ^e^	13.28 ± 0.16 ^c^	13.76 ± 0.15 ^b^	12.84 ± 0.12 ^d^
Arginine	10.23 ± 0.11 ^a^	9.11 ± 0.12 ^e^	9.82 ± 0.08 ^c^	10.11 ± 0.18 ^b^	9.53 ± 0.12 ^d^
Proline	7.35 ± 0.04 ^a^	6.58 ± 0.04 ^e^	7.01 ± 0.08 ^c^	7.24 ± 0.08 ^b^	6.82 ± 0.12 ^d^
ΣEAA	78.29	68.63	74.29	76.95	71.60
ΣNEAA	111.27	97.82	106.33	109.15	102.62
TAA	189.56	166.45	180.62	186.10	174.22
EAA/NEAA	0.704	0.702	0.699	0.705	0.698
EAA/TAA (%)	41.30	41.23	41.13	41.35	41.10

Values are expressed as mean ± standard deviation (*n* = 3). Different superscript letters within a row indicate significant differences (*p* < 0.05).

**Table 3:** Effect of different processing methods on phenolic compound content in dry weight (DW).

Compound	Raw Leaves	Boiling	Microwaving	Steaming	Stir-Frying
Phenolic acids (mg/100 g DW)					
Gallic acid	1.55 ± 0.08 ^a^	0.88 ± 0.06 ^e^	1.48 ± 0.08 ^c^	1.51 ± 0.11 ^b^	0.91 ± 0.08 ^d^
Protocatechuic acid	8.71 ± 0.12 ^a^	5.58 ± 0.16 ^e^	8.55 ± 0.18 ^c^	8.62 ± 0.20 ^b^	5.65 ± 0.22 ^d^
Vanillic acid	4.37 ± 0.08 ^a^	3.11 ± 0.06 ^e^	4.08 ± 0.12 ^c^	4.27 ± 0.12 ^b^	3.15 ± 0.08 ^d^
Chlorogenic acid	5.72 ± 0.12 ^a^	3.94 ± 0.08 ^c^	5.63 ± 0.12 ^b^	5.67 ± 0.14 ^b^	3.99 ± 0.08 ^c^
Caffeic acid	75.33 ± 2.20 ^a^	33.62 ± 1.18 ^e^	73.11 ± 2.26 ^c^	74.28 ± 2.12 ^b^	34.51 ± 1.48 ^d^
p-Coumaric acid	2.19 ± 0.08 ^a^	1.49 ± 0.06 ^e^	2.05 ± 0.04 ^c^	2.11 ± 0.04 ^b^	1.52 ± 0.08 ^d^
Ferulic acid	31.44 ± 0.64 ^a^	22.62 ± 0.40 ^d^	30.76 ± 0.34 ^c^	31.19 ± 0.20 ^b^	22.73 ± 0.40 ^d^
Sinapic acid	29.57 ± 0.82 ^a^	14.22 ± 0.14 ^e^	28.58 ± 0.28 ^c^	29.17 ± 0.20 ^b^	15.34 ± 0.16 ^d^
Flavonoids (mg/100 g DW)					
Rutin	28.15 ± 0.42 ^a^	21.04 ± 0.24 d	27.78 ± 0.22 b	26.23 ± 0.26 ^c^	20.45 ± 0.44 ^e^
Quercetin	11.22 ± 0.28 ^a^	9.44 ± 0.14 d	10.97 ± 0.16 c	11.03 ± 0.22 ^b^	9.21 ± 0.11 ^e^
Isoquercitrin	7.76 ± 0.22 ^a^	3.98 ± 0.012 ^e^	7.11 ± 0.31 ^b^	5.62 ± 0.12 ^c^	5.11 ± 0.11 ^d^
Astragalin	9.22 ± 0.44 ^a^	5.46 ± 0.62 ^e^	8.89 ± 0.32 ^b^	7.85 ± 0.42 ^c^	7.14 ± 0.42 ^d^
Kaempferol	16.17 ± 1.4 ^a^	10.31 ± 1.24 ^d^	15.22 ± 1.62 ^c^	15.78 ± 1.23 ^b^	9.56 ± 0.82 ^e^
Myricetin	8.15 ± 0.43 ^a^	5.12 ± 0.41 ^e^	8.03 ± 0.61 ^b^	5.91 ± 0.42 ^d^	6.32 ± 0.42 ^c^

Values are expressed as mean ± standard deviation (*n* = 3). Means within a column followed by different superscript letters are significantly different (*p* < 0.05).

**Table 4:** HPLC-DAD validation parameters of phenolic compounds.

Compound	λ (nm)	tR (min)	Calibration Curve	R^2^	LOD	LOQ	Precision (%RSD)	Recovery (%)
Gallic acid	280	3.2	y = 12543x + 81	0.9989	0.03	0.10	2.8	95.6
Protocatechuic acid	280	5.1	y = 11321x + 246	0.9992	0.04	0.13	3.5	97.1
Vanillic acid	280	7.3	y = 10455x+ 42	0.9987	0.05	0.16	2.4	94.8
Chlorogenic acid	320	10.5	y = 13221x + 335	0.9996	0.07	0.22	1.9	101.3
Caffeic acid	320	12.8	y = 14567x + 395	0.9993	0.05	0.17	2.1	98.6
p-Coumaric acid	320	15.6	y = 12110x + 275	0.9988	0.06	0.19	3.8	96.0
Ferulic acid	320	18.2	y = 13890x + 360	0.9991	0.06	0.20	2.6	99.4
Sinapic acid	320	20.1	y = 12987x + 342	0.9985	0.08	0.25	4.2	93.8
Rutin	360	22.4	y = 15678x + 510	0.9995	0.08	0.24	1.7	100.6
Quercetin	360	25.3	y = 14980x + 470	0.9997	0.06	0.19	1.5	99.2
Isoquercitrin	360	23.8	y = 14210x + 455	0.9990	0.09	0.28	2.9	96.9
Astragalin	360	24.6	y = 13750x + 421	0.9986	0.10	0.31	3.4	95.2
Kaempferol	360	27.2	y = 15230x + 505	0.9994	0.06	0.20	2.2	98.7
Myricetin	360	21.9	y = 14890x + 488	0.9988	0.08	0.26	3.1	97.3

tR—apparent retention time; LOD—limit of detection (S/N = 3); LOQ—limit of quantification (S/N = 10); RSD—relative standard deviation.

**Table 5:** Antioxidant activity of raw and processed *C. maxima* leaves.

Processing Method	TPC (mg GAE/100 g DW)	TFC (mg QE/100 g DW)	Total Carotenoids (mg/100 g DW)	FRAP (mmol Fe^2+^/100 g DW)	DPPH (mg TE/100 g DW)	ABTS (mg TE/100 g DW)
Raw leaves	482.3 ± 18.5 ^a^	177.1 ± 8.2 ^a^	12.8 ± 0.5 ^a^	3.54 ± 0.12 ^c^	153.65 ± 7.14 ^b^	88.12 ± 4.24 ^b^
Boiling	398.4 ± 15.3 ^d^	98.1 ± 5.6 ^d^	8.2 ± 0.4 ^d^	2.78 ± 0.10 ^e^	88.17 ± 4.22 ^e^	64.21 ± 2.88 ^e^
Microwaving	451.6 ± 17.2 ^b^	128.5 ± 6.4 ^c^	10.9 ± 0.5 ^b^	3.88 ± 0.15 ^b^	149.7 ± 6.18 ^c^	84.17 ± 4.15 ^c^
Steaming	476.0 ± 19.1 ^a^	171.9 ± 7.4 ^b^	12.2 ± 0.4 ^a^	4.12 ± 0.16 ^a^	155.12 ± 6.96 ^a^	91.17 ± 5.42 ^a^
Stir-frying	425.2 ± 16.8 ^c^	102.1 ± 5.8 ^d^	9.4 ± 0.3 ^c^	3.42 ± 0.11 ^d^	121.19 ± 4.66 ^d^	73.21 ± 5.20 ^d^

Values are expressed as mean ± standard deviation (*n* = 3). Means within a column followed by different superscript letters are significantly different (*p* < 0.05).

**Table 6:** Concentration-dependent α-amylase inhibitory activity (%) of raw and thermally processed *C. maxima* leaf extracts compared with acarbose.

Concentration (mg/mL)	Raw Leaves	Stir-Frying	Microwaving	Steaming	Boiling	Acarbose
0.05	4.02 ± 0.41	3.11 ± 0.37	7.86 ± 0.52	11.21 ± 0.63	2.08 ± 0.31	24.18 ± 0.95
0.10	31.84 ± 1.08	24.96 ± 0.92	29.72 ± 1.04	35.31 ± 1.10	15.88 ± 0.82	58.34 ± 1.42
0.15	49.67 ± 1.26	39.85 ± 1.14	45.83 ± 1.19	52.18 ± 1.28	34.91 ± 1.03	79.12 ± 1.67
0.20	54.83 ± 1.39	45.94 ± 1.20	51.74 ± 1.27	57.86 ± 1.41	41.87 ± 1.09	92.36 ± 1.88

Values are expressed as mean ± standard deviation (*n* = 3).

**Table 7:** Effect of culinary processing on antinutritional compounds of *C. maxima* leaves.

Compound	Raw Leaves	Boiling	Microwaving	Steaming	Stir-Frying
Antinutritional compounds (mg/g DW)					
Phytates	81.05 ± 0.17 ^a^	16.62 ± 0.12 ^e^	34.58 ± 0.14 ^d^	65.34 ± 0.15 ^b^	44.33 ± 0.22 ^c^
Oxalates	85.46 ± 0.05 ^a^	15.56 ± 0.11 ^e^	35.68 ± 0.18 ^c^	64.44 ± 0.06 ^b^	29.58 ± 0.09 ^d^
Tannins	42.34 ± 0.12 ^a^	21.25 ± 0.04 ^e^	29.35 ± 0.31 ^d^	33.43 ± 0.16 ^b^	30.62 ± 0.06 ^c^
Apparent retention on a dry-weight basis (%)					
Phytates	100.0	20.5	42.7	80.6	54.7
Oxalates	100.0	18.2	41.8	75.4	34.6
Tannins	100.0	50.2	69.3	79.0	72.3

Values are expressed as mean ± standard deviation (*n* = 3). Means within a row followed by different superscript letters are significantly different (*p* < 0.05).

## Data Availability

The original contributions presented in this study are included in the article. Further inquiries can be directed to the corresponding authors.
